# Cognitive Tomography Reveals Complex, Task-Independent Mental Representations

**DOI:** 10.1016/j.cub.2013.09.012

**Published:** 2013-11-04

**Authors:** Neil M.T. Houlsby, Ferenc Huszár, Mohammad M. Ghassemi, Gergő Orbán, Daniel M. Wolpert, Máté Lengyel

**Affiliations:** 1 Computational and Biological Learning Lab, Department of Engineering, University of Cambridge, Cambridge CB2 1PZ, UK; 2 Wigner Research Institute for Physics, Hungarian Academy of Sciences, Budapest 1121, Hungary; 3 Department of Cognitive Science, Central European University, Budapest 1023, Hungary

## Abstract

Humans develop rich mental representations that guide their behavior in a variety of everyday tasks. However, it is unknown whether these representations, often formalized as priors in Bayesian inference, are specific for each task or subserve multiple tasks. Current approaches cannot distinguish between these two possibilities because they cannot extract comparable representations across different tasks [1–10]. Here, we develop a novel method, termed cognitive tomography, that can extract complex, multidimensional priors across tasks. We apply this method to human judgments in two qualitatively different tasks, “familiarity” and “odd one out,” involving an ecologically relevant set of stimuli, human faces. We show that priors over faces are structurally complex and vary dramatically across subjects, but are invariant across the tasks within each subject. The priors we extract from each task allow us to predict with high precision the behavior of subjects for novel stimuli both in the same task as well as in the other task. Our results provide the first evidence for a single high-dimensional structured representation of a naturalistic stimulus set that guides behavior in multiple tasks. Moreover, the representations estimated by cognitive tomography can provide independent, behavior-based regressors for elucidating the neural correlates of complex naturalistic priors.

## Highlights

• Cognitive tomography estimates complex prior distributions across different tasks
• Humans have highly structured and idiosyncratic priors over faces
• Priors for faces are task independent
• The estimated priors allow prediction of behavior for novel stimuli and tasks

## Graphical Abstract

**Figure:**
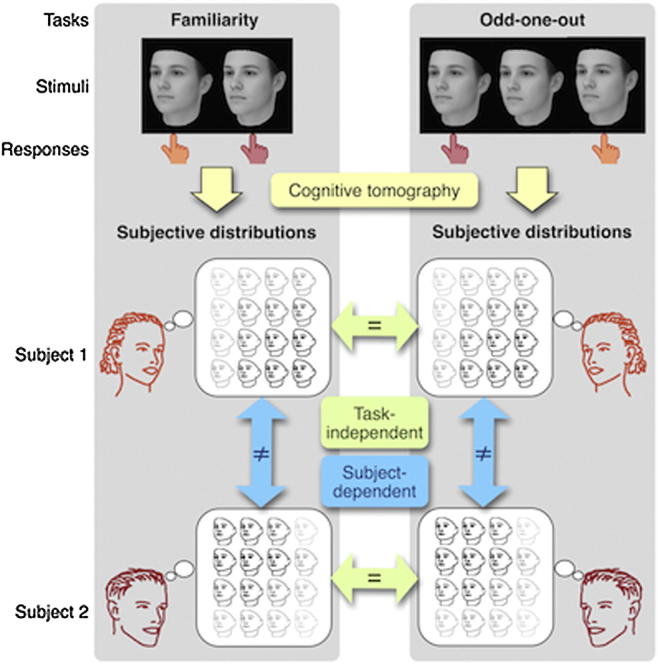

## Results

Human performance in a wide range of individual perceptual tasks has been shown to be close to that of an ideal observer that combines sensory evidence with prior expectations according to the rules of Bayesian inference [11]. Moreover, many perceptual illusions have been shown to arise from the influence of priors in the face of sensory uncertainty or ambiguity [12]. Thus, characterizing priors for natural stimuli and understanding how they are used is central to the study of human perception.

The priors we use for simple one-dimensional variables, such as speed of movement for visual objects [3] or direction of sunlight [13], have each been carefully characterized in the context of a specific perceptual task. However, surprisingly little is known about the nature of priors for complex, high-dimensional real-life stimuli, such as faces, and whether such priors depend on the task in which they are employed. The task dependence of priors, in particular, addresses a fundamental assumption of the Bayesian paradigm that has so far gone untested: to allow for efficient learning and powerful generalization, natural priors should be shared across tasks such that the same prior can be used in many different situations, predicting task independence. Conversely, demonstration of a prior in only a single task leaves open the possibility that the behavioral effects attributed to that prior are instead caused by idiosyncratic response strategies elicited by the task and thus the real prior may be different from that assumed [14, 15]. In order to test the task independence of priors, we need to compare the priors used in different tasks that operate on the same stimulus set. To do so requires us to overcome a major obstacle: the lack of any method for extracting potentially complex, high-dimensional priors for naturalistic stimuli across different tasks.

### Cognitive Tomography

Here we develop a novel Bayesian approach, cognitive tomography, that can be applied to a wide variety of behavioral tasks by allowing simple discrete choices to be used to reveal detailed and quantitative information about a subject’s personal, potentially complex and high-dimensional mental representations. The term “cognitive tomography” is motivated by the isomorphism with traditional structural tomography in which a detailed high-dimensional physical structure is reconstructed from a sequence of low-dimensional measurements (derived from mathematical integrals over the underlying structure) by solving the “inverse problem” [16]. Analogously, our method reconstructs an individual subject’s representational structure using a sequence of simple discrete choices (arising from mathematical integrals over the underlying structure) by explicit inversion of a model describing how responses depend on mental representations.

**Figure 1:**
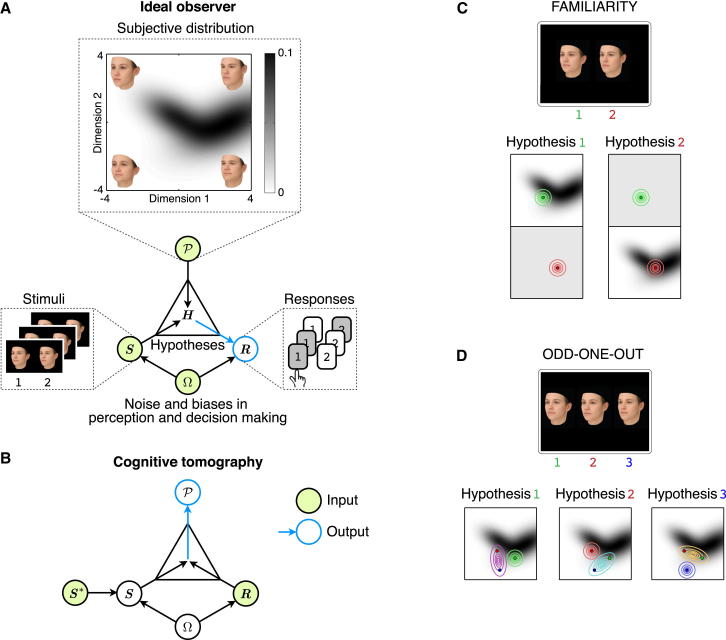
Cognitive Tomography Applied to Estimating Priors for Faces (A) Information flow in the ideal observer model. In the model, a subjective distribution, P, encodes prior knowledge about stimuli. In this study, a subjective distribution for faces assigns a probability value (gray levels) to each face as a function of its location in feature space (here the two dimensions of the feature space correspond to the first two principal components of the structure of faces [19] and are measured in units of SD). Representative faces corresponding to the corners of the feature space are shown. The ideal observer infers hypotheses, ***H***, about the stimuli it perceives, ***S***, using prior knowledge encoded in P. Based on the inferred hypotheses, it computes the final response ***R***. Both perception and decision making are subject to noise and biases, Ω. (B) Cognitive tomography inverts the ideal observer model to compute P based on ***R*** and the presented stimuli, ***S^∗^***, which is corrupted by perceptual noise to yield ***S***. Note that information available to the ideal observer and cognitive tomography (circles with green fill) to compute their final output (blue arrows and circles) is complementary. (C) In the familiarity task, participants are presented with a pair of faces (top) and are required to pick the one that they judge more familiar. Each face corresponds to a particular location in feature space (colored dots in the bottom panels correspond to stimuli in the top panels). The ideal observer model makes its choice by considering two hypotheses (bottom; hypothesis 1, face 1 is more familiar than face 2; hypothesis 2, vice versa) that each specify a way in which the stimuli could have been generated. According to these hypotheses, the familiar face is a sample from the subjective distribution (corrupted by perceptual noise; colored covariance ellipses), and the unfamiliar face is sampled randomly and uniformly from the feature space (also subject to perceptual noise). Given a subjective distribution and the covariance of perceptual noise, the ideal observer assigns a probability to each hypothesis and then through a decision process (also including noise) determines the probability of each possible response. (D) In the odd-one-out task, participants are presented with three faces and are required to pick the one that looks the most different from the other two (top). Each hypothesis corresponds to two of the faces being noise-corrupted versions (bottom; pairs of dots enclosed by covariance ellipses) of the same underlying face (centers of ellipses) and the third face (the odd one out) being a noisy version of a truly different face (isolated dots within covariance ellipses, here shown as circles). See also Figure S1 for further details and validation of the method.

We start with the idea that objects can be described by multidimensional features, and a subject’s prior over a class of objects is a probability distribution over those features [17, 18]. For example, the feature space we use is based on the physical appearance of a large sample of human faces scanned in three dimensions and is constructed along the first two principal components of their geometrical structure [19]. Figure 1A (top) shows this feature space as well as the prior of a hypothetical subject plotted in this space: gray scale indicates the probability, according to the subject, with which a face represented by each location belongs to the class of familiar faces. To avoid terminological confusion later, we will refer to a subject’s prior as their “subjective distribution,” and in line with other studies of perceptual priors, we assume that it affects perceptual decisions without necessarily being explicitly accessible by the subject. The key element of our approach is that we explicitly treat the subjective distribution as an unknown quantity that cannot be observed directly and thus needs to be inferred from observable behavior. For this, we use “ideal observer” models that link subjective distributions to behavior, and by inverting these models using probabilistic machine learning methods [20] we estimate the subjective distribution.

Ideal observer models formalize subjects’ responses in simple perceptual decision-making tasks as a two-step process [21] (Figure 1A; see also the Supplemental Experimental Procedures available online). First, the subject performs Bayesian inference to compute the probability of different hypotheses, ***H***, about how the perceived stimuli, ***S***, may have arisen within the context of the given task, based on prior knowledge about these stimuli encoded in their subjective distribution, P. Then, the subject gives a response based on the probabilities of these hypotheses, where the decision-making process itself may also be imperfect such that the subject does not always produce the response which corresponds to the most probable hypothesis. The result of this two-step process is a probability distribution over possible responses, ***R***, given the presented stimuli, the subjective distribution, and other parameters of the ideal observer model, Ω, such as noise and biases in perception and decision making:

(1) $$P_{\text{ideal}\;\text{observer}}\left(R|S,P,\Omega \right).$$

The essence of our method (Figure 1B) is to use a second layer of Bayesian inference to invert the ideal observer model in order to estimate the subjective distribution from the set of responses the subject gives to the stimuli presented over the course of an experiment, ***S^∗^***. Due to perceptual noise, the stimuli perceived by the subject, ***S***, are not exactly the same as the stimuli they are presented with ***S^∗^***, and the experimenter only knows (and controls) the latter. Thus, this uncertainty needs to be taken into account as a probability distribution over the subject’s perceived stimuli given the presented stimuli and noisiness in the subject’s perception, $P\left(S|S^{*},\Omega \right)$. We place flexible prior distributions over both the subjective distribution, P(P), and the parameters describing perceptual and decision making noise and biases, P(Ω). Using Bayes’ rule, we compute the posterior distribution over possible subjective distributions by combining these priors with the ideal observer model as the likelihood (and integrating out the other parameters):

(2) $$P\left(P|R,S^{*}\right)\propto P\left(P\right)\int d\Omega \;P\left(\Omega \right)\int dS\;P\left(S|S^{*},\Omega \right)\;P_{\mathrm{ideal}\;\mathrm{observer}}\left(R|S,P,\Omega \right).$$

Crucially, while the ideal observer is task-specific by definition, the subjective distribution need not be. Thus, this separation in our model between these two parts allows us to analyze behavioral data from different tasks and quantify the relation between the derived subjective distributions.

We applied cognitive tomography to infer subjective distributions in two different tasks. In one task, subjects had to decide which of two faces was more familiar (Figure 1C), while in the other task they were asked to choose which of three faces was the odd one out (OOO; Figure 1D). Therefore, the requirements in these two tasks were fundamentally different: the familiarity task explicitly asked subjects to judge each stimulus in terms of its familiarity, with no requirement to compare the structure of the two faces, while the OOO task required subjects to compare the structures of the three faces to each other, without the need to determine their familiarity. Importantly, by using ideal observer models, our mathematical framework allowed us to treat these tasks in a unified formalism even though they had different task requirements and were different at a psychological level.

In the familiarity task, we modeled the ideal observer as comparing directly the probabilities that the subjective distribution assigned to the two faces and choosing the one with the higher probability (Figure 1C, the face on the right being more familiar). Thus, this model does not necessarily imply that subjects simply judge familiarity based on averageness: in fact, if the prior is multimodal, or nonconvex (as is the case in Figure 1A), then its “average” might have low probability density and hence our model would predict a low familiarity rating for it. In order to make this ideal observer model conceptually consistent with that of the OOO task (see below), we reformulated the same decision rule in terms of the ideal observer comparing the probabilities of different hypotheses about how the stimuli might have arisen [6, 22]. Each hypothesis posited that one of the faces was a sample from the subject’s subjective distribution (Figure 1C, dots), with some potential perceptual noise added (Figure 1C, ellipses), while the other face came from another distribution (here assumed to be uniform; see also Figure S1 and the Supplemental Experimental Procedures for a decision theoretic rationale).

In the OOO task, our ideal observer model entertained three hypotheses, each positing that two of the displayed stimuli were noisy realizations of the same underlying face which was sampled from the subjective distribution (Figure 1D, dots within the same elongated ellipse), while the third, the odd one out, was a noisy realization of another face, corresponding to another sample from the subjective distribution. Thus, for stimuli that are equidistant from each other (as in 90% of trials in our experiment), the three hypotheses can only be distinguished using the subjective distribution. While in general the influence of the subjective distribution can be complex, one simple intuition is based on considering the two possible ways in which a subject can account for any apparent differences when presented with two stimuli. They either attribute these differences to just perceptual noise (while assuming that only one object was sampled from their subjective distribution), and thus deem the two stimuli to be identical at a fundamental level, or they assume that the differences between the stimuli are due to there having been two different objects sampled from their subjective distribution, and thus that the two stimuli are really different. As the two accounts differ in the number of objects sampled from the subjective distribution (one or two, respectively), their relative likelihood is scaled by the probability of the stimuli under the subjective distribution: the higher this probability is, the more likely the second account becomes, resulting in a higher propensity to discriminate stimuli that are closer to high probability regions of the subjective distribution. With three stimuli present, as in our OOO task, it is one out of such a high probability pair that will likely be the odd one out (i.e., hypothesis 1 or 2 in Figure 1D; see also Figure S1).

In both the familiarity and the OOO task, the behavioral response of the subject was modeled as comparing the probabilities of the different hypotheses and making a choice based on these probabilities, with noise and biases in the perceptual and decision making processes so that less probable hypotheses were sometimes chosen. We validated the method to show that it is able to extract subjective distributions from such noisy responses and is robust to the choice of feature space and test stimuli (Figure S1).

### Complex, Task-Invariant Subjective Distributions over Faces

**Figure 2:**
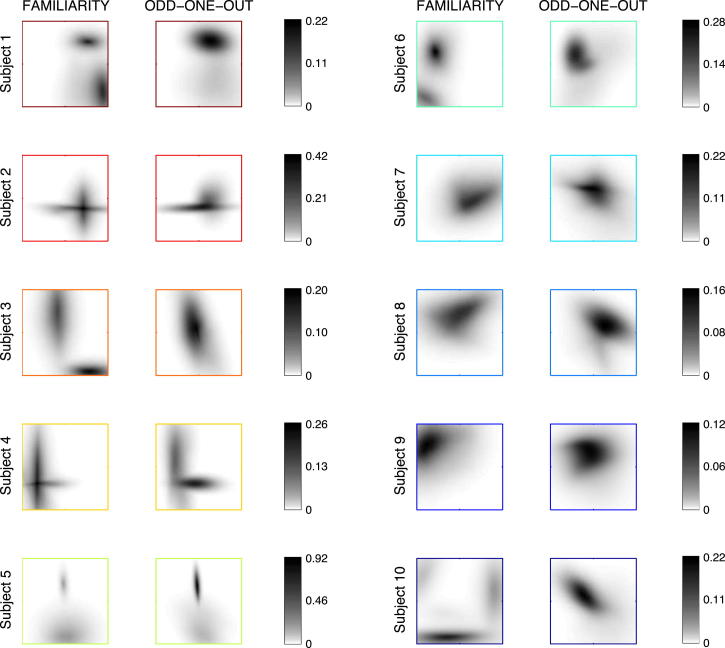
Subjective Distributions Inferred from the Two Tasks for the Ten Subjects Each plot shows the probability (gray levels) over the principal component feature space (±4 SD along each dimension as in Figure 1A). Subjects are ordered according to their consistency score (from high to low), which is a model-free measure of the repeatability of their behavior for identical stimuli. See also Figure S2 for inferred values of other decision parameters.

We extracted the subjective distributions of ten subjects who performed both the familiarity and the OOO task. The subjective distributions were independently estimated in each subject and in each task. The distributions we found were complex, often not well described by a single mode, and varied greatly across subjects (Figures 2 and S2). This variation across subjects in the familiarity task confirms that subjects were performing this task by judging familiarity as intended, with respect to prior experience with faces in the world rather than based on familiarity with respect to the stimulus distribution presented in the experiment [23]—as unlike the extracted subjective distributions, the stimulus distribution was identical across subjects.

**Figure 3:**
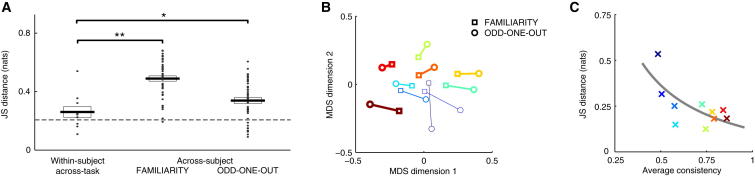
Comparison of Subjective Distributions Across Tasks and Subjects (A) Jensen-Shannon (JS) distances between subjective distributions inferred in the same subject for the two different tasks (left), inferred in different subjects within the familiarity (middle) and odd-one-out (right) tasks. Dots show individual comparisons (left, subjects; middle and right, subject pairs), boxes show mean ± SE. The dashed line shows the estimated lower bound based on the average distance between distributions inferred from two halves of the data from the same task and same subject. ^∗^p < 0.05, ^∗∗^p < 0.01. (B) Two-dimensional embedding of subjective distributions for the ten subjects and two tasks (symbols) based on multidimensional scaling applied to all 190 pairwise JS distances. Lines connect distributions of the same subject, and line width is proportional to the consistency score of the subject. (C) Across-task JS distances for each subject (symbols) against the subject’s task-average consistency score. The regression line shows hyperbolic fit to data. Colors for subjects in (B) and (C) are as in Figure 2. See also Figure S3.

Importantly, despite differing greatly across subjects, subjective distributions were similar between tasks within each subject. In order to quantify dissimilarities between subjective distributions, we computed a standard information theoretic measure of distance between them, the Jensen-Shannon (JS) divergence. JS divergences between distributions corresponding to the same subject but to different tasks were significantly lower than JS divergences between the distributions of different subjects within each task (Figure 3A, p = 5 × 10^−5^ and p = 0.047 in the familiarity and OOO tasks, respectively). Embedding of all subjective distributions in a two-dimensional space by multidimensional scaling [1] based on their JS distances also showed that subjective distributions strongly clustered based on subject and not task (Figure 3B).

The apparent differences between the estimated priors of some of our subjects across the two tasks could have arisen either because priors are truly different or because of randomness in subjects’ responding (accounted for in our model by perceptual and decision noise; Figure S2) that makes the estimation less accurate. However, as we had repeated a fraction of the trials, we were able to quantify the consistency of subjects by measuring the probability that they gave the same answer to the same stimuli on different occasions [24]. This provided us with an independent model-free measure of the reliability of subjects. We found that, as expected because of subjects’ perceptual uncertainty and behavioral stochasticity, consistency scores were far from 100% (familiarity, 0.76 ± 0.04; OOO, 0.62 ± 0.05; mean ± SE). Importantly, the subjective distributions of the more consistent subjects were also more similar in the two tasks (Figure 3C, r = 0.69, p = 0.028; see also Figure 2, in which subjects are ordered from most to least consistent, and Figure S3). This suggests that within-subject dissimilarities of estimated subjective distributions are due to factors not related to the stimuli and the corresponding priors, but to inherent variability in subjects’ responses.

### Predicting Behavior Within and Across Tasks

**Figure 4:**
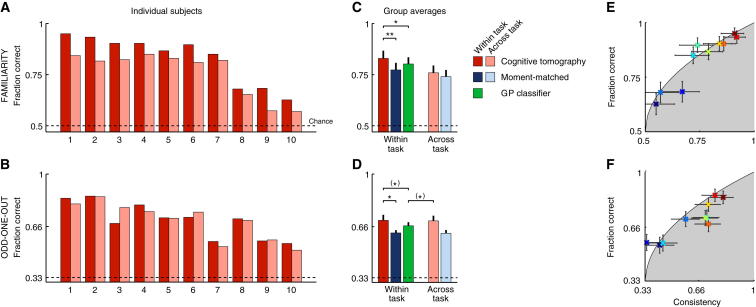
Predicting Subjects’ Responses Within and Across Task with Different Models (A and B) Individual subjects. Performance of cognitive tomography is shown for within-task (red) and across-task predictions that is using subjective distributions inferred from one task to predict behavior in the other task (pink). The dashed line shows chance performance. Subjects are ordered by their average consistency on the two tasks (as in Figure 2). (C and D) Group averages (mean ± SE) comparing cognitive tomography (red and pink bars) to alternative predictors. Replacement of subjective distributions with moment-matched Gaussians, thus ignoring the fine structural details of the subjective distributions, decreases performance (dark blue, within task; light blue, across task). A Gaussian process (GP) classifier that is directly optimized to fit subjects’ stimulus-to-response mappings without assuming the existence of subjective distributions also performs worse and is unable to generalize across tasks (green bars). ^(^^∗^^)^p < 0.10, ^∗^p < 0.05, ^∗∗^p < 0.01. (E and F) Within-task predictive performance of cognitive tomography for each subject (symbols color coded as in Figure 2) against their consistency levels. Boundary of gray shaded area shows expected upper bound on the performance of any predictor as a function of consistency. Error bars show 95% confidence intervals. See also Figure S4 for a more detailed analysis of predictive performance.

If indeed the subjective distributions we inferred are fundamental to subject’s mental representations, then they should allow us to predict subjects’ responses to novel stimuli. Moreover, if the subjective distributions are truly task independent, we should be able to predict behavior in one task based on the subjective distribution we inferred from behavior on the other task. Figure 4 shows that both within- and across-task predictions (red and pink bars, respectively) are significantly above chance (dashed line; p = 1.1 × 10^−5^ and p = 4.9 × 10^−5^ for within- and across-task predictions for the familiarity task [top row], respectively; p = 2.7 × 10^−6^ and p = 4.8 × 10^−6^ for within- and across-task predictions for the OOO task [bottom row], respectively; see also Figure S4). Remarkably, within-task predictions for the familiarity task are very close to an expected upper bound that can be computed based on subjects’ consistency [25] (Figures 4E and 4F). Furthermore, the subjective distributions we extracted from the familiarity task also provided across-task predictions in the OOO task that were as accurate as within-task predictions in that task (p = 0.84). This suggests that the familiarity task is an efficient paradigm for extracting priors which generalize to other tasks (although it may not be readily applicable to all perceptual domains, such as visual motion).

We used three alternative models for predicting subjects’ responses to validate the results that we obtained by cognitive tomography. First, the assumption that the two tasks invoked intrinsically different decision rules was tested through the use of the same decision rule in the OOO task as in the familiarity task: simply choosing the most familiar face, or conversely the least familiar face, as the odd one out. Both of these decision models had significantly poorer predictive performance than the original decision model; in fact, their performance was sometimes close to chance (Figure S4). This confirms that subjects processed the same set of stimuli in fundamentally different ways in the two tasks.

Second, although the subjective distributions in Figure 2 show a great deal of structural detail, it could be that these fine details are idiosyncratic and have little relevance for subjects’ behavior. We sought to rule out this possibility by replacing each inferred subjective distribution with a distribution that lacked these fine structural details but had the same mean and covariance (a single moment-matched Gaussian). If the structural details of the distribution we inferred were idiosyncratic, then predictions based on the simplified “moment-matched” distributions should be as good as those based on the inferred distributions. However, taking into account the originally inferred subjective distributions led to significantly better predictions than using the moment-matched distributions (Figures 4C and 4D, blue bars; p = 0.0056 and p = 0.025 in the familiarity and OOO tasks, respectively; see also Figure S4). This shows that the details of the subjective distributions revealed by our inference algorithm, which go beyond simple means and covariances, rather than being artifactual have true behavioral relevance.

Third, to test whether predicting subjects’ responses benefits from assuming that there is a task-independent component of their mental representation, we predicted responses using a Gaussian process (GP) classifier that is a state-of-the-art learning algorithm that has no notion of subjective distributions and is optimized directly for within-task prediction. Nevertheless, our method outperforms the GP classifier (Figure 4C and 4D, green bars; p = 0.023 and p = 0.076 in the familiarity and OOO tasks, respectively; see also Figure S4). Importantly, the GP classifier directly fits subjects’ stimulus-to-response mappings without extracting underlying subjective distributions and thus has no way to provide across-task predictions. In contrast, in the OOO task, even our across-task predictions are as good as (even marginally better, p = 0.092, than) the within-task predictions of the GP classifier algorithm.

## Discussion

Previous methods aimed at extracting mental representations were limited because they were constrained to be used with only one particular task [1–10]. For example, multidimensional scaling can be used to construct a psychological space in which the proximity of individual stimuli is determined by the subject’s similarity judgments (akin to the judgments subjects needed to make in our OOO task) [1], but it is unclear how this space could be useful to process or predict familiarity judgments about the same stimuli. Similarly, reverse correlation methods can be used to extract a classification image in a task that essentially requires familiarity judgments [7, 25], but such a classification image only provides information about the mean or mode of the prior [26] and thus remains uninformative about the rich structural details of the priors we have demonstrated. Moreover, it is again unclear how the classification image could be relevant to similarity judgments in tasks such as our OOO task, especially given that we have shown familiarity not to be directly predictive of behavior in the OOO task. In contrast, our method extracts detailed subjective distributions over multidimensional feature spaces in a way that it can be used with essentially any task type in which performance depends on these distributions.

The priors we extracted were strikingly different across subjects but invariant across tasks. The distinct subject specificity of the priors for faces we found is in contrast with lower-level sensory priors which have been found to be more similar across subjects [3]. However, even such lower-level priors, for example those over the direction of illumination [13] and the speed of visual motion [23], have been shown to be plastic to experience. Thus, the difference between our subjects’ priors over faces may in part reflect their different personal experiences with faces, possibly relating to their geographical and cultural backgrounds. Personal experiences for lower-level features can be expected to be more uniform, which could account for the similarity of the priors for such features across subjects in other studies.

The issue of task invariance is also important because task-specific and -independent representations map onto two fundamental mechanisms of learning: discriminative and generative. In discriminative learning, one learns the mapping from stimuli to responses directly for each task with the aim of optimizing task performance. Thus, discriminative learning is solely tailored to improve performance in each specific task separately. In contrast, in generative learning, one learns the probability of experiencing different stimuli irrespective of the task. This task-independent representation can then be used to generate different stimulus-response mappings depending on task demands. Classical theories of learning suggest that task-independent representations, arising through generative learning, are beneficial when the range of tasks is wide, and hard to prespecify. For example, generative representations of low-level perceptual features such as edges in visual scenes account well for neural and behavioral data [27–29]. In particular, behavior in tasks that only rely on such low-level features has been shown to use different readout mechanisms operating on representations that are shared across tasks [30]. However, when the set of required tasks is limited or is well known a priori, task-specific representations, brought about by discriminative learning, would be beneficial [31]. For example, discriminative learning would be expected for high-level tasks such as object recognition and categorization [32–35]. This theoretical distinction makes our results of task-independent representations of human faces particularly unexpected because this is a domain in which there is a set of naturally required tasks (such as familiarity, categorization, and outlier detection) for which learning might be expected to be specialized. Therefore, one might expect that other representations, for which the human brain may have less specialized circuitry [36, 37], will also be task independent.

Our results thus suggest that there should be a common neural underpinning of a subject’s priors employed across several tasks. This is not a conclusion that could have been easily achieved through neuronal recordings from higher-order cortical areas because it would require inverting a model that defines how these subjective distributions are reflected in neural activity. While there are well-established ideal observer models that describe how prior distributions are reflected in subjects’ behavior, there is no comparable understanding of how complex, multidimensional priors are reflected in neuronal firing [11, 38]. However, our cognitive tomography method is directly applicable to search for such neural correlates as it provides a method for computing an independent, purely behavior-based regressor for techniques such as functional imaging and neurophysiology. Moreover, our method can be readily generalized beyond the domain of perception, for example, to estimate conceptually abstract priors such as over moral beliefs by modeling subjects’ responses to questionnaires using ideal observer models derived from item response theory [39]. Thus, in combination with neural recording techniques, our work opens the way to the study of the neural underpinning of even such abstract priors.

## Supplemental Information

Document S1. Supplemental Experimental Procedures and Figures S1–S4
