# Supplementary material for: Cognitive Tomography Reveals Complex, Task-Independent Mental Representations
Source: Curr Biol. 2013 Nov 4;23(21):2169–75. doi: 10.1016/j.cub.2013.09.012 (PMC3898796; doi:10.1016/j.cub.2013.09.012)
Supplement: Document S1. Supplemental Experimental Procedures and Figures S1–S4 [file mmc1.pdf]

Current Biology, Volume *23*

Supplemental Information

Cognitive Tomography Reveals Complex Task-Independent Mental Representations

Neil M.T. Houlsby, Ferenc Huszár, Mohammad M. Ghassemi, Gergő Orbán, Daniel M. Wolpert, and Máté Lengyel

Supplemental Figures and Legends

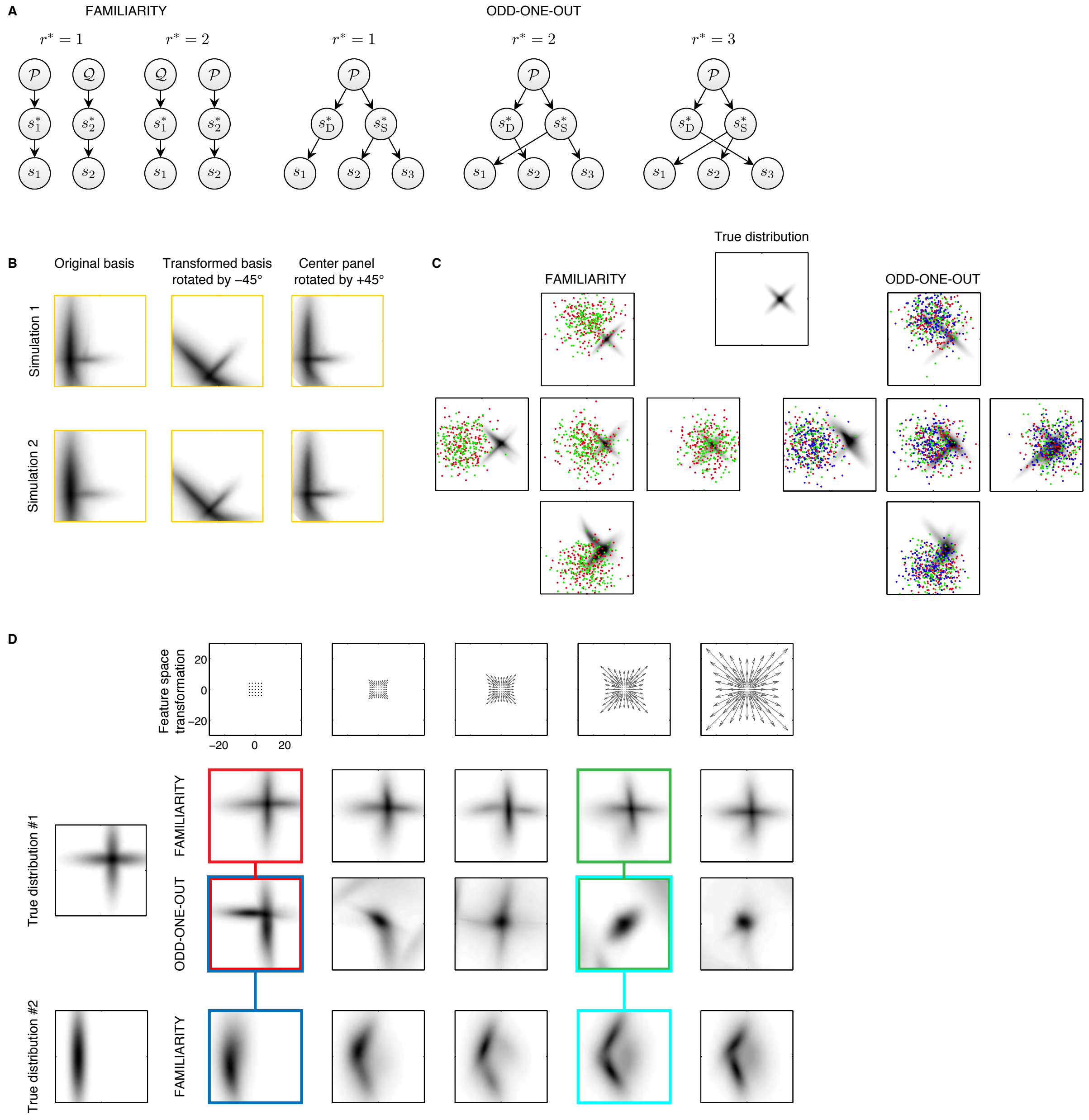

Figure S1. Model details and validation of cognitive tomography; related to Figure 1. *(continued on next page)*

Figure S1. Model details and validation of cognitive tomography; related to Figure 1. *(continued from preceding page)*
(A) Graphical models of the hypotheses (corresponding to different values of r^*) in the ideal observer models of the two tasks (left: familiarity, right: odd-one-out). Circles denote random variables, arrows denote conditional independence relationships.
(B) Left: Repeated runs of the MCMC sampler on the same data set yield near-identical subjective distributions. Subjective distribution of an example subject is shown (subject 4 of Fig. 2). Note that the characteristic fine structure of the subjective distribution is retained upon repeated inference on the same data. Center: Rotating the stimulus dimensions does not affect the inference algorithm. Inference of the subjective distribution was performed in a basis of stimulus features that was rotated by $-45°$ from the original basis around the center of the feature space. Cardinal directions of the subjective distribution are rotated together with the basis. Right: For ease of comparison with subjective distributions on the left, subjective distributions in the center were rotated back by $+45°$. Gray-levels are as in Fig. 2.
(C) Inferred subjective distributions are robust to changes in the distribution of stimuli used in the experiments. Synthetic subjective distribution (top, center) used to generate responses in the familiarity (left) and odd-one-out tasks (right). Stimuli (colored dots) and inferred subjective distributions (gray-levels) using five different stimulus distributions (individual panels). Green and red dots for the familiarity task represent stimuli displayed on the left and right of the screen, respectively; green, red, and blue dots for the odd-one-out task represent stimuli displayed on the left, in the middle, and on the right of the screen, respectively. Mean of the stimulus distribution was $(0, 0)$, $(2, 0)$, $(-2, 0)$, $(0, -2)$, and $(0, 2)$, while keeping the standard deviation constant (1.5). Main qualitative characteristics of the subjective distribution were retained despite substantial changes in the stimulus distribution.
(D) Nonlinear warping of the feature space – or, equivalently, non-Gaussian and non-translation-invariant perceptual noise – is unlikely to result in artifactual similarity between subjective distributions inferred in the same subject from different tasks. Synthetic subjective distributions (left) were used to generate responses under increasingly nonlinear warping transformations of the feature space (top row), determining the mapping from presented to perceived stimuli. Inference of subjective distributions was performed without taking the warping into account, as would be the case with real subjects for whom the warping is unknown (bottom three rows). Similar distributions are only inferred when the warping is weak and the underlying subjective distribution for the two tasks is the same (red boxes). Dissimilar distributions are found with strong warping (e.g. green boxes), and when the underlying distributions are different either with weak (blue boxes) or strong warping (cyan boxes).

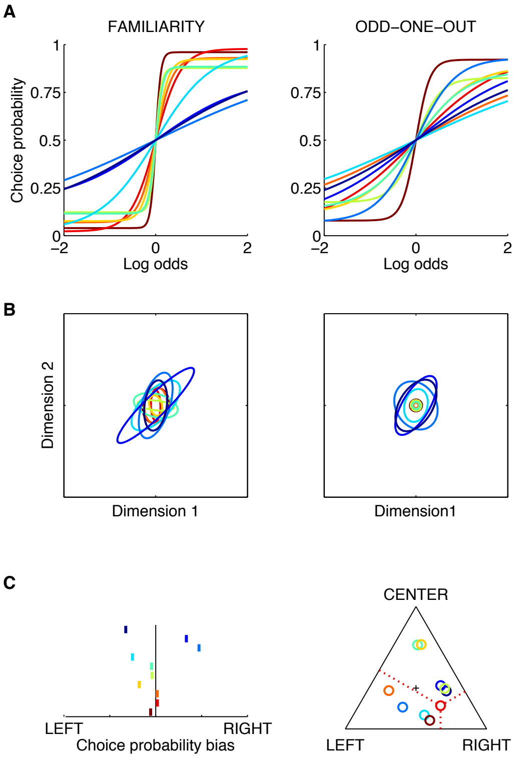

Figure S2. Posterior mean estimates of nuisance parameters of the ideal observer model in the familiarity (left) and OOO task (right); related to Figure 2.

(A) Sigmoidal decision functions for each subject: inverse decision noise, β, is the slope of the sigmoid at 0, and lapse rate, κ, is the offset of its lower and upper bounds from 0 and 1, respectively. Note that while in the familiarity task the soft-max decision rule (Eq. S9) is formally equivalent to a logistic sigmoidal function of the log odds of the two alternative hypotheses (abscissa), in the OOO task this is not the case (because there are three alternative hypotheses). Nevertheless, the two parameters have analogous meanings in the two tasks, and thus they are visualised here through sigmoidal functions in both cases to aid intuition and across-task comparison.

(B) Covariance ellipses characterising perceptual noise, Σ_{noise}, for each subject. Surrounding box shows principal component feature space (± 4 s.d. along each dimension as in Figs. 1A and 2).

(C) Prior decision biases, π, for each subject shown as points along a unit segment (familiarity, vertical offsets only for visibility) or within an equilateral triangle (OOO). Endpoints of the segment (familiarity) and vertices of the triangle (OOO) correspond to decision biases only favoring the corresponding choice (i.e. prior decision bias of 1 for that choice), other locations represent a linear interpolation of these biases (i.e. no biases for the point at the centre). In all panels, colors for subjects are as in Fig. 2.

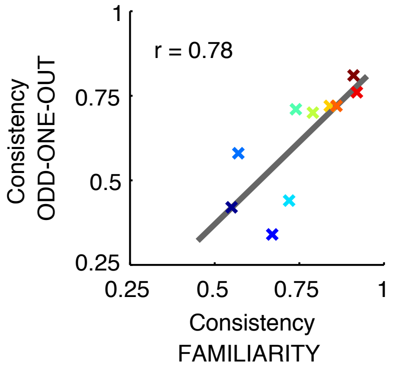

Figure S3. Consistencies in the two tasks; related to Figure 3.
Consistency scores measured in the two tasks were highly correlated with each other (*$p<0.05$), and also with the natural logarithm of the inverse decision noise parameters, $\ln \beta$, of the subjects (familiarity: $r=0.75$, $p=0.013$; OOO: $r=0.60$, $p=0.048$, data not shown). Colors for subjects are as in Fig. 2.

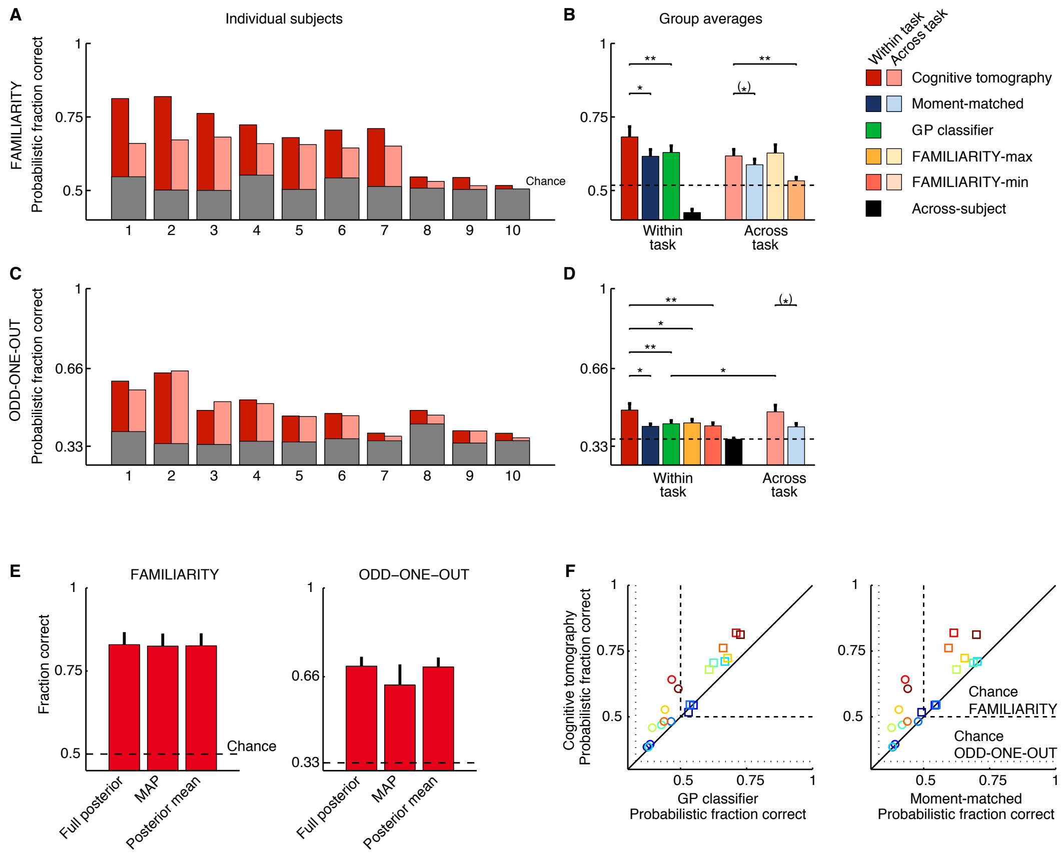

Figure S4. Comparison of predictions with alternative methods; related to Figure 4. *(continued on next page)*

Figure S4. Comparison of predictions with alternative methods; related to Figure 4. *(continued from preceding page)*
(A-D) Predicting subjects' responses within and across task with different models – using the probabilistic fraction correct measure of predictive performance. Bars show fraction of correctly predicted responses based on cross validation. Predictive performances are shown for individual subjects (A,C) and across subjects (B,D, mean $\pm$ s.e.) in the familiarity (A,B) and odd-one-out task (C,D).
(A,C) Performance of cognitive tomography is shown for within-task (red) and across-task predictions (pink). Gray bars show individual chance levels (section 6.6). Subjects are ordered by their average consistency on the two tasks (as in Figs. 2 and 4).
(B,D) Comparing cognitive tomography (red and pink bars) to alternative predictors. Replacing subjective distributions with moment-matched Gaussians (section 6.4.1), thus ignoring the fine structural details of the subjective distributions, decreases performance (dark blue: within-task, light-blue: across-task). A Gaussian process (GP) classifier that is directly optimized to fit subjects' stimulus-to-response mappings without assuming the existence of subjective distributions (section 6.4.2) also performs worse and is unable to generalize across tasks (green bars). Using ideal observer models that assume that subjects respond on the odd-one-out task as if they were performing the familiarity task (section 6.4.3) also leads to significantly worse performance (dark and light orange / salmon: within- and across task predictions for choosing the most / least familiar stimulus as the odd one out, respectively), thus confirming the fundamentally different nature of the two tasks. Finally, predicting responses based on other subjects' subjective distributions (black bars) also degrades performance substantially. Dashed lines show average of subject-specific chance levels. $^{(*)}p<0.10$, $^{*}p<0.05$, $^{**}p<0.01$.
(E) Comparison of predictive performance (mean $\pm$ s.e.) based on Bayesian integration, MAP estimation, and using the posterior mean estimate for the familiarity task (left) and the odd-one-out task (right). No significant differences were found between the performance of different methods ($p>0.1$ for all pair-wise comparisons).
(F) Cognitive tomography outperforms the GP classifier (left) and the moment-matched Gaussian (right): within-task prediction of the responses of individual subjects (circles and squares for the odd-one-out and familiarity tasks, respectively) was above chance (dashed lines) in all cases but one (the exception was subject 10: moment-matched Gaussian was 0.2% below chance on the familiarity task), and the performance of cognitive tomography was consistently higher than that of the GP classifier (left, 18/20 symbols are above the diagonal) and the moment-matched Gaussian (right, 19/20 symbols are above the diagonal). Colors for subjects are as in Fig. 2.

Supplemental Experimental Procedures

1 Experiments

Ten participants (7 male, 3 female, age range 21–41, mean 27.8), who were naive to the purpose of the experiment gave their informed consent and participated in the study. All had normal, or corrected to normal vision. The study was approved by the Psychology Research Ethics Committee of the University of Cambridge. Subjects participated in two tasks in which they made judgments about faces presented on a computer screen.

Subjects sat approximately 60 cm in front of a 18-inch LCD monitor (resolution 1280$\times$1024 pixels, refresh rate of 75 Hz). Three dimensional photo-realistic faces were generated using the Basel Face Model (BFM) [S1] and rendered at 300$\times$300 pixels. The BFM is based on 3D scans of 200 faces (half male and half female) to each of which a mesh (with over 50,000 vertices) is fit. Principal Components Analysis (PCA) is performed separately on the three-dimensional coordinates and on the colors of the vertices. Faces can then be reconstructed as a combination of the 199 principal components. For the experiments we varied the first two principal components of the structure within ± 4 standard deviations around the means (zero), while leaving all other principal components (including those determining color) fixed at their mean values.

1.1 The feature space used in this study

In our method, as in other related work [S2, S3], the feature space itself needs to be predefined. We chose a feature space spanned by the (first two) principal components of the structural face-space defined by Ref. [S1] which thus reflects the 'natural statistics' of faces. (This space should not be confused with that spanned by so-called 'eigenfaces', conventionally used in studies of human face perception [S4]. Eigenfaces are principal components of the pixel values of grayscale images of faces, whereas our features are principal components of the geometrical structure of 3D faces. One way to illustrate the fundamental difference between the two is that in our space, realistic faces can be generated by using only two dimensions, i.e. setting all but two coordinates to zero, while this would be impossible using eigenfaces.) Our choice was motivated by the assumption that subjects' representation of faces would be using a feature space that is adapted to the natural statistics of faces, as such an adaptation has been demonstrated to be a fundamental principle underlying the organization of lower-level sensory representations [S5].

While there is no guarantee that the feature space we chose is the one actually used by subjects, as long as there is a smooth mapping between the two spaces, the subjective distributions we extract can still be analysed (Fig. S1D). Although the choice of feature dimensions by itself does not bias our inferences about subjective dimensions, our priors over the subjective distribution, and our choice of a translation-invariant perceptual noise distribution (see below) are obviously specific to this feature space and will thus inevitably bias the inference procedure (as would any other model choice do). Nevertheless, these biases remain benign: the high predictive power of our method (Figs. 4 and S4) indicates that salient features of subjects' mental representations are well captured by the feature space we chose. Moreover, the task-invariance of inferred distributions also indicates that our feature space is appropriate (Fig. S1D). Also note that we make no assumptions that subjects use all pixels of the image; they could attend to different parts of the image, such as the mouth or the nose. However, as these features vary smoothly with the principal components we use to parametrize faces, our method is still applicable and the results can still be meaningfully plotted in

our feature space.

We chose a two-dimensional feature space as a compromise between having a large enough space that can accommodate a wide variety of subjective distributions but does not require an excessive number of trials to estimate those distributions. Importantly, the inference algorithm itself readily generalizes to higher dimensional spaces. However, as the number of dimensions increases the number of trials needed to infer subjective distributions also increases in our current paradigm which samples stimuli randomly for each trial (see below). Therefore extensions to high dimensions will require active learning paradigms [S6] which can substantially reduce the number of trials by selecting stimuli on each trial for which knowing the subject's response will be maximally informative about their subjective distribution.

1.2 The familiarity task

For the familiarity task, subjects were given the following instructions: 'For this test we will show you two faces. Choose the face which is more familiar to you'. On each trial, two faces were displayed horizontally adjacent on the screen (Fig. 1C). At the start of each trial, the mouse cursor was positioned midway between the two faces and subjects used the mouse to make their choice by clicking on one of the faces.

We generated stimuli by first drawing a 'center' location from a bivariate, isotropic zero-mean Gaussian with a standard deviation of 3, $\mathbf{c} \sim \mathcal{N}(\mathbf{0}, 3\mathbf{I})$. The two stimuli were then generated by first sampling a unit vector with a uniformly random orientation, $\mathbf{v} = \begin{pmatrix} \cos\alpha \\ \sin\alpha \end{pmatrix}$ where $\alpha \sim \mathcal{U}(0, 2\pi)$, and making the two stimuli to be symmetrical around the 'centre' in the direction of this vector with a distance of 1.5: $\mathbf{s}_1 = \mathbf{c} + 0.75\,\mathbf{v}$, $\mathbf{s}_2 = \mathbf{c} - 0.75\,\mathbf{v}$. Any stimulus lying outside the range of ± 4 along either dimension was redrawn from an isotropic zero-mean Gaussian with a standard deviation of 3, truncated at ± 4 along each dimension. This procedure led to around 56% of samples with a separation of 1.5, and the remainder had a range of separations. (The fine details of the distribution of stimuli used in the experiments did not matter, as our method for inferring mental representations is robust to them, see Fig. S1C.)

Subjects performed 1000 trials with a short break every 100 trials. In the last 100 trials we repeated the pairs of stimuli presented during the first 100 trials with the locations of stimuli and the order of trials randomized. This allowed us to assess each subject's consistency (see section 6.5).

1.3 The odd-one-out task

For the odd-one-out task (OOO) task, subjects were given the following instructions: 'For this test we will show you 3 faces. Two people are from country A, one person is from country B. During each trial, click on the person from country B, the odd one out.' Subjects were presented with three horizontally arranged faces (Fig. 1D), and chose the odd one out by clicking on the appropriate face.

For each trial, we generated faces by first drawing a centre point $\mathbf{c}$ from an isotropic zero-mean Gaussian with a standard deviation of 3, truncated at ± 3.5 along each dimension. The three faces were selected to lie at the vertices of a triangle with a uniformly random orientation. For the first 100 trials we used isosceles triangles with the length of the longer sides being 1.5 and the length of shorter side gradually increasing over these trials from 0.5 to 1.5 to yield equilateral triangles from trial 100 onwards. The first 100 trials eased the subjects into the task as two of the faces were clearly similar compared to the third face. As in the

familiarity task, subjects performed 1000 trials and the last 100 trials repeated the stimulus triplets presented during trials 101-200.

Each participant completed the odd-one-out task (75±35 mins) followed by the familiarity task (155±33 mins). By running subjects on the odd-one-out followed by the familiarity task, we avoided a potential confusion due to a carry-over of the instructions that would have led subjects to simply choose the most or least familiar of the three faces rather than the odd one out. We also explicitly tested for the possibility of following familiarity response rules in the odd-one-out task in the behavior of our subjects and found no evidence for it (Fig. S4). Conversely, the instructions for the odd-one-out task cannot be used in the familiarity task.

2 Ideal observer models

We denote the set of stimuli perceived by the subject in trial t by $S^{(t)}$, and the subject's response to this stimulus by $r^{(t)}$. In our model, a subject's decision is principally governed by their subjective distribution that we denote by $\mathcal{P}$. The subjective distribution, $\mathcal{P}$, is mathematically a probability distribution over stimuli in feature space (two dimensional in our experiments). We assume that the subjective distribution does not change during the course of the experiment and that the subject's responses, $r^{(t)}$, are independent and identically distributed given the stimuli, $S^{(t)}$, and their subjective distribution, $\mathcal{P}$.

The stochastic dependence of the subject's response, $r^{(t)}$, on the stimuli, $S^{(t)}$, and subjective distribution, $\mathcal{P}$, is described as a probability distribution $\mathrm{P}\big(r^{(t)}|S^{(t)},\mathcal{P}\big)$. We derive this dependence from *ideal observer models* (and drop trial index t to simplify notation). An ideal observer model computes the statistically optimal decision strategy given the subject's mental representation of stimuli, $\mathcal{P}$, and what they know about the task. In particular, the ideal observer bases its decision on Bayesian inference over what the best response in each trial would be. From the subject's perspective, the stimuli $S^{(t)}$ and the subjective distribution $\mathcal{P}$ are observed. Each of the possible responses correspond to a different hypothesis, $r^{*(t)}$, about how the current stimuli were generated. The subject's task is to determine the posterior probability that each of these hypotheses may be correct:

$$\mathrm{P}(r^* = i|S,\mathcal{P}) \propto \pi_i \cdot \mathrm{P}(S|\mathcal{P}, r^* = i) \tag{S3}$$

The posterior is a product of two terms. First, subjects may have a preference for choosing stimuli at particular screen locations, which we model as a prior bias, π_i, for believing that hypothesis i is the correct one, and hence response i should be given. Second, this prior needs to be combined with the likelihood, $\mathrm{P}(S|\mathcal{P}, r^* = i)$, that defines the probability with which the combination of perceived stimuli are expected given the subjective distribution and that hypothesis i is correct.

Importantly, the functional form of this likelihood term depends on the particular psychophysical task the subject is solving. In principle, any psychophysical task can be given an ideal observer model description, and such an ideal observer model could be readily used in our framework.

2.1 The familiarity task

In the case of the familiarity task, the pair of stimuli perceived by the subject in a trial S are described as a pair of two dimensional vectors, $S = \{s_1, s_2\}$, where s_1 and s_2 are the feature space representation

of the face displayed on the left and right of the screen, respectively. Following Refs. [S3, S7], there are two alternative hypotheses (Figs. 1C and S1A) that may explain the stimuli, and the subject has to decide which one is correct. Under hypothesis $r^* = 1$, the left-hand stimulus s_1 is familiar, the right-hand one, s_2 is unfamiliar, and vice versa for $r^* = 2$. From the perspective of an ideal observer entertaining $\mathcal{P}$ as its subjective representation of stimuli, a familiar stimulus is sampled from $\mathcal{P}$; an unfamiliar stimulus can be arbitrary, thus it is sampled from an (improper) uniform distribution, $\mathcal{Q}$, over stimuli. Furthermore, both stimuli are corrupted by perceptual noise, described by the distribution $\mathcal{O}(s; s^*)$ defining the probability of perceiving s as a noise-corrupted version of the true stimulus presented by the experimenter, s^*.

Therefore, the likelihoods of the two hypotheses become:

$$\mathrm{P}(S = \{s_1, s_2\} \,|\mathcal{P}, r^* = 1) = \int \mathcal{O}(s_1; s_1^*)\ \mathcal{P}(s_1^*)\ \mathrm{d}s_1^* \cdot \int \mathcal{O}(s_2; s_2^*)\ \mathcal{Q}(s_2^*)\ \mathrm{d}s_2^* \tag{S4}$$

$$\mathrm{P}(S = \{s_1, s_2\} \,|\mathcal{P}, r^* = 2) = \int \mathcal{O}(s_1; s_1^*)\ \mathcal{Q}(s_1^*)\ \mathrm{d}s_1^* \cdot \int \mathcal{O}(s_2; s_2^*)\ \mathcal{P}(s_2^*)\ \mathrm{d}s_2^* \tag{S5}$$

where s^* is the true stimulus, uncorrupted by perceptual noise and thus not directly observable for the subject. (The integral over the perceptual noise distribution for the stimulus sampled from $\mathcal{Q}$ could obviously be omitted, as the marginal distribution of s obtained after this integral is still just an improper uniform, but it is included here for symmetry.)

Although we choose the alternative distribution $\mathcal{Q}$, from which unfamiliar stimuli are assumed to be sampled, to be uniform, we could choose a more flexible form and infer it from data via the same procedure that we use to estimate $\mathcal{P}$. However, by choosing it to be uniform we ensure that the resulting decision rule is intuitive (because it simply amounts to comparing directly the probabilities that the subjective distribution assignes to the two stimuli) and consistent with the Luce choice rule (see below).

2.2 The odd-one-out task

In each trial of the odd-one-out task the subject perceives three stimuli $S = \{s_1, s_2, s_3\}$. Accordingly, the subject entertains three hypotheses, each corresponding to one of the stimuli being the odd one out. Under hypothesis $r^* = 1$, two of the stimuli, s_2 and s_3 are related, whilst the first stimulus s_1 is unrelated to them. Following Refs. [S8, S9], we can formalize the similarity or relatedness of s_2 and s_3 as being noise-corrupted realizations of the same underlying stimulus s_{S}^*, which is sampled from the distribution $\mathcal{P}$. The odd face, s_1, is a potentially noise-corrupted version of a different stimulus s_{D}^* which is also sampled from $\mathcal{P}$, but independently of s_{S}^*. Fig. 1D illustrates this generative process, S1A shows the graphical models corresponding to the three hypotheses.

Under this generative process the likelihoods of the three hypotheses are:

$$\mathrm{P}(S = \{s_1, s_2, s_3\} \,|\mathcal{P}, r^* = 1) = \int \mathcal{O}(s_1; s_{\mathrm{D}}^*)\, \mathcal{P}(s_{\mathrm{D}}^*)\, \mathrm{d}s_{\mathrm{D}}^* \int \mathcal{O}(s_2; s_{\mathrm{S}}^*)\, \mathcal{O}(s_3; s_{\mathrm{S}}^*)\, \mathcal{P}(s_{\mathrm{S}}^*)\, \mathrm{d}s_{\mathrm{S}}^* \tag{S6}$$

$$\mathrm{P}(S = \{s_1, s_2, s_3\} \,|\mathcal{P}, r^* = 2) = \int \mathcal{O}(s_2; s_{\mathrm{D}}^*)\, \mathcal{P}(s_{\mathrm{D}}^*)\, \mathrm{d}s_{\mathrm{D}}^* \int \mathcal{O}(s_1; s_{\mathrm{S}}^*)\, \mathcal{O}(s_3; s_{\mathrm{S}}^*)\, \mathcal{P}(s_{\mathrm{S}}^*)\, \mathrm{d}s_{\mathrm{S}}^* \tag{S7}$$

$$\mathrm{P}(S = \{s_1, s_2, s_3\} \,|\mathcal{P}, r^* = 3) = \int \mathcal{O}(s_3; s_{\mathrm{D}}^*)\, \mathcal{P}(s_{\mathrm{D}}^*)\, \mathrm{d}s_{\mathrm{D}}^* \int \mathcal{O}(s_1; s_{\mathrm{S}}^*)\, \mathcal{O}(s_2; s_{\mathrm{S}}^*)\, \mathcal{P}(s_{\mathrm{S}}^*)\, \mathrm{d}s_{\mathrm{S}}^* \tag{S8}$$

This model of 'generative similarity' has been shown to account for a wide range of experimental data

on subjective judgments of similarity [S9], including generalization gradients that match data better than Shepard's classical theory [S10].

3 Choice probabilities

In the previous sections we have derived how the posterior distribution over possible responses being correct, $\mathrm{P}(r^* = i|S, \mathcal{P})$, is computed. We complete our model by specifying how the subject's actual response, r, is related to this posterior.

If subjects behaved statistically optimally by trying to minimize the number of false decisions they make (appropriate in an nAFC task, when their utility function uniformly penalizes all responses that are not the correct one), they would always choose the response corresponding to the hypothesis with the highest posterior probability $\mathrm{P}(r^* = i|S, \mathcal{P})$ (maximum a posteriori, or MAP decision). We introduce a generalization of simple MAP decision making which is a standard and more flexible model of decision making [S3, S7, S11] allowing for stochasticity in the decision process and lapses of attention:

$$\mathrm{P}(r = i|S, \mathcal{P}) = (1-\kappa)\,\frac{\mathrm{P}(r^* = i|S, \mathcal{P})^{\beta}}{\sum_{j=1}^{R} \mathrm{P}(r^* = j|S, \mathcal{P})^{\beta}} + \frac{\kappa}{R} \tag{S9}$$

where R denotes the number of possible responses ($R = 2$ for familiarity, $R = 3$ for odd-one-out), and β and κ are parameters that jointly control how deterministic the subject's decisions are. Parameter κ describes stimulus-independent decision noise and can be interpreted as the lapse rate: on κ fraction of trials there is a lapse of attention and the subject responds randomly. Parameter β describes stimulus-dependent decision noise: by setting how hard the threshold is for choosing different responses depending on the posterior probabilities of their corresponding hypotheses. Larger values of β result in more deterministic behavior and for $\kappa = 0$ and in the limit $\beta \to \infty$ the decision strategy approaches the deterministic MAP strategy; for $\beta = 1$ the subject performs probability matching by selecting each response in proportion with the posterior probability for the underlying hypothesis; and $\beta = 0$ corresponds to random decision making.

4 Perceptual noise

A final source of suboptimality in a subject's behavior is perceptual noise which we took into account for computing the likelihoods of the competing hypotheses (see previous two sections). For simplicity, we assumed this noise to be Gaussian distributed, $\mathcal{O}(s; s^*) = \mathcal{N}(s; s^*, \Sigma_{\text{noise}})$, centered on the true stimulus s^* with covariance Σ_{noise}. Although, in principle, other perceptual noise distributions would be possible, our choice is motivated by two reasons. First, we chose a translation-invariant distribution (i.e. one in which only the mean depends on s^*) to keep the number of parameters constrained and to avoid non-identifiability issues when jointly inferring the subjective distribution and perceptual noise parameters. Second, the particular Gaussian shape is practical, because we model the subjective distribution as a mixture of Gaussians and thus the convolution with Gaussian perceptual noise (Eqs. S4-S5 and S6-S8) can be performed in closed form. Although we expect both choices to be eventually incorrect, in the context of the current study they seemed to have resulted in an acceptable approximation (Fig. S1D).

Perceptual noise also plays another important role in our formalism. The ideal observer is defined in terms of the stimuli the subject *perceives*, S, whereas the experimenter only has access to (and control over) the stimuli that the subject is *presented with*, S^*. Thus, in order to be able to define response probabilities conditioned on the presented stimuli, this uncertainty about the unobserved perceived stimuli needs to be marginalized out using the same perceptual noise distribution, $\mathcal{O}(s; s^*)$:

$$\mathrm{P}(r = i|S^*, \mathcal{P}) = \int \mathrm{d}S \prod_j \mathcal{O}\big(s_j; s_j^*\big) \ \mathrm{P}(r = i|S, \mathcal{P}) \tag{S10}$$

However, performing this integral would be computationally prohibitive, and the effects are phenomenologically very closely matched by a simpler model which, instead of performing this integral, directly uses Eq. S9 conditioned on the presented rather than the perceived stimuli with decreased β (and increased κ) to capture the increased apparent stochasticity of responding (not shown). Thus, we used this simpler approximation and note that the interpretation of the perceptual and decision noise parameters is ambiguous because decision noise in this version of the model captures in part the effects of perceptual noise. Since the values of these nuisance parameters were not of primary interest in this study (and were eventually integrated out to avoid overfitting and to obtain the best estimate for the subjective distribution – see section 5.2), we regarded this acceptable. Were the actual values of nuisance parameters relevant, Eq. S10 would need to be used, in conjunction with a psychophysical paradigm specifically designed to disentangle the effects of perceptual and decision noise.

In sum, parameters π, β, κ, and Σ_{noise} may vary across subjects, therefore we treat them as unobserved quantities and infer them from experimental data, together with the subjective distribution $\mathcal{P}$.

5 Inverting ideal observer models by Bayesian inference

The ideal observer models of the two tasks provide a probabilistic description of subjects' responding based on their subjective distribution $\mathcal{P}$, and additional 'nuisance' parameters, $\Omega = \{\pi, \beta, \kappa, \Sigma_{\text{noise}}\}$: their prior biases π_i, decision noise β, lapse rate κ, and perceptual noise Σ_{noise}. For brevity, we denote all parameters that collectively govern a subject's responding as $\theta = \{\mathcal{P}, \pi, \beta, \kappa, \Sigma_{\text{noise}}\}$. We now have $\mathrm{P}\Big(r^{(t)} = i|S^{*(t)}, \theta\Big)$, that we can interpret as a forward model of decision making. For inferring parameters θ from the subject's responses we need to invert this forward model by using Bayes' rule (see below for a description of the prior distribution over parameters, $\mathrm{P}(\theta)$):

$$\mathrm{P}\Big(\theta|\{S^{*(t)}, r^{(t)}\}_{t=1}^T\Big) = \frac{\prod_t \mathrm{P}\Big(r^{(t)}|S^{*(t)}, \theta\Big) \ \mathrm{P}(\theta)}{\int \prod_t \mathrm{P}\Big(r^{(t)}|S^{*(t)}, \theta'\Big) \ \mathrm{P}(\theta') \ \mathrm{d}\theta'} \tag{S11}$$

5.1 Parameter priors

Bayesian inference requires defining the prior distribution, $\mathrm{P}(\theta)$, that expresses our prior beliefs about parameters θ. We defined independent and minimally informative priors on each of the free parameters separately:

Subjective distribution $\mathcal{P}$ was parametrized as a mixture of $K = 4$ multivariate normal distributions $\mathcal{P}(s) = \sum_{i=1}^{K} w_i \mathcal{N}(s; \mu_i, \Sigma_i)$, described by parameters w_i, μ_i, and Σ_i. This family of distributions is flexible enough to capture complex probability distributions, but analytically convenient and simple enough for computations to be carried out efficiently.

We parametrized the weights as $w_i = {e^{w'_i}}/{\sum_{j=1}^{K} e^{w'_j}}$ to ensure they were positive and summed to one, with $w'_i \sim \mathcal{N}(0, 1)$, and for the other parameters we had priors $\mu_i \sim \mathcal{N}(\mathbf{0}, \mathbf{I})$ and $\Sigma_i \sim$ Wishart$(\mathbf{I}, 3)$. To ensure covariance matrices were positive definite and to improve numerical stability of the inference algorithm we used the lower-triangular Bartlett decomposition [S12].

Decision noise β was constrained to be non-negative, parametrized as $\beta = e^{\beta'}$ with $\beta' \sim \mathcal{N}(0, 1)$.

Lapse rate κ was bounded between 0 and 1, $\kappa \in [0, 1]$, and parametrized as $\kappa = {1}/{1+e^{-\kappa'}}$ with $\kappa' \sim \mathcal{N}(0, 1)$

Perceptual noise covariance Σ_{noise} had a Wishart prior: $\Sigma_{\text{noise}} \sim$ Wishart$(\mathbf{I}, 3)$, implemented via the Bartlett decomposition [S12].

Prior decision bias π was a discrete distribution over $R = 2$ and $R = 3$ responses in the familiarity and odd-one-out task, respectively, parametrized as $\pi_i = {e^{\pi'_i}}/{\sum_{j=1}^{R} e^{\pi'_j}}$, with $\pi'_i \sim \mathcal{N}(0, 1)$.

5.2 Sampling algorithm

Having defined these prior distributions and using the ideal observer-based likelihood $\mathrm{P}\left(r^{(t)} | S^{*(t)}, \theta\right)$ we can now use Bayes' rule to calculate the posterior $\mathrm{P}\left(\theta | \{S^{*(t)}, r^{(t)}\}_{t=1}^{T}\right)$ (Eq. S11). However, the parameters space over which the posterior needs to be computed is large, and moreover, the integral in the denominator of Equation S11, called the marginal likelihood, is intractable. Therefore we used a Markov chain Monte Carlo (MCMC) sampling algorithm to generate samples from the posterior. In particular, as both the likelihood and the prior were differentiable with respect to parameters θ we used hybrid Monte Carlo [S13] to collect 10,000–50,000 samples (each including 20 leapfrog steps), with the results of the first 25% of the steps discarded as 'burn-in'.

The result of MCMC was a sequence $\theta_1, \ldots, \theta_N$ of $N \in$ [10,000, 50,000] samples that were distributed as $\mathrm{P}\left(\theta | \{S^{*(t)}, r^{(t)}\}_{t=1}^{T}\right)$. Of the components of θ we were particularly interested in the subjective distribution $\mathcal{P}$ and considered the other parameters, Ω, as nuisance parameters (we show posterior mean estimates of these parameters in Fig. S2). All quantities of interest that we plotted and quantified depended on integrals over the posterior distribution which were approximated by averages over the samples produced by the MCMC algorithm (except where otherwise noted, see Fig. S4E). In particular, integrating out the nuisance parameters, Ω, was important because they were partially unidentifiable (see section 6.3.3), and their interpretability was limited (see section 4), and also because we wanted to avoid overfitting.

5.3 Validation of the inference algorithm

MCMC is a non-deterministic procedure, therefore repeated runs are variable, possibly producing different results. However, after a sufficiently large number of steps, moments of the sequence converge, and we should not see any difference in multiple runs. Thus, as a basic test of the validity of our sampling algorithm,

we ran four independent chains for each dataset. Figure S1B shows for a randomly chosen subject the posterior mean subjective distribution obtained from two independent runs of MCMC.

The particular prior distribution we have chosen over subjective distributions is invariant under unitary transformations of the coordinate system in which we describe stimuli. Therefore if we transform the coordinates describing stimuli in any dataset and perform inference, we should obtain subjective distributions that are transformed accordingly, provided that the transformation is unitary. We therefore rotated the stimulus coordinates by $45°$, and performed inference. These results are shown in Figure S1B. The fact that inference is unaffected by such transformations demonstrates that the algorithm is robust to whether the experimenter's choice of feature coordinates corresponds to the feature dimensions relevant for the subject.

A factor that may confound the subjective distributions we infer from behavior is the distribution of stimuli presented to the subject during the experiment: if we never observe the subject making decisions about stimuli around a localized region in stimulus space, we cannot expect to recover their subjective distribution accurately in that region. To demonstrate that the results of inference were robust to changes in the stimulus distribution, we conducted an experiment in which the subjective distribution was known and fixed whilst the stimulus distribution was varied. Data was generated randomly based on a synthetic subjective distribution by simulating an ideal observer model, and our inference algorithm was used to recover the subjective distribution from each set of simulated responses. The inferred subjective distributions were qualitatively similar even for minimally overlapping stimulus distributions (Fig. S1C).

Another potential confounding factor may be a mismatch between the feature space we use to define the coordinates of the stimuli and the space used by the subject internally to represent these stimuli (see also section 1.1). This is equivalent to perceptual noise, which is assumed by our method to be translation-invariant Gaussian (see section 4), being non-Gaussian and non-translation-invariant. However, simulations with synthetic subjects show that the high degree of within-subject similarity of inferred distributions that we observed across tasks is unlikely to be obtained unless our feature space is approximately correct (ie. perceptual noise is approximately translation-invariant Gaussian) and the underlying distributions are truly similar (Fig. S1D).

6 Details of data analyses

6.1 The posterior mean subjective distribution

In Figures 2 and S1B-D we visualize the posterior mean subjective distribution, which is obtained by computing the mean probability assigned to any particular stimulus s under the posterior:

$$\bar{\mathcal{P}}(s) = \int \mathcal{P}(s)\ \mathrm{P}\Big(\theta | \{S^{*(t)}, r^{(t)}\}_{t=1}^{T}\Big)\ \mathrm{d}\theta \tag{S12}$$

Note that although each individual sample from the posterior over $\mathcal{P}$ is a mixture of $K = 4$ Gaussians, when taking the mean of these samples, the resulting distribution will be a mixture of $4N$ Gaussians, where N is the number of samples used. Therefore the posterior mean subjective distributions can take almost arbitrarily complex shapes, and can for example have sub-Gaussian tails.

6.2 Jensen-Shannon divergence

Figure 2 allows one to visually compare the recovered subjective distributions and assess how similar or dissimilar they are to each other. These dissimilarities were quantified more rigorously by using the Jensen-Shannon (JS) divergence, which is defined over a pair of probability distributions P and Q as follows:

$$\mathrm{JS}[P\|Q] = \frac{1}{2}\,\mathrm{KL}\left[P\middle\|\frac{P+Q}{2}\right] + \frac{1}{2}\,\mathrm{KL}\left[Q\middle\|\frac{P+Q}{2}\right] \tag{S13}$$

where $\mathrm{KL}[P\|Q]$ denotes the Kullback-Leibler (KL) divergence defined as

$$\mathrm{KL}[P\|Q] = \int P(s)\,\log\frac{P(s)}{Q(s)}\,\mathrm{d}s \tag{S14}$$

The JS divergence has several useful properties that make it suitable for our analysis. It is zero if and only if the two distributions are identical, and it is always finite and bounded from above by 1. Unlike the KL divergence, it is also symmetric in its arguments, and its square-root is a metric between probability distributions (that is, beside its aforementioned properties it also satisfies the triangle inequality).

Unfortunately, the JS divergence between Gaussian mixture distributions – which is what we used to parametrize subjective distributions – can not be expressed in closed analytical form. However, we may compute an approximation to it by discretizing stimulus space and computing the JS divergence between the discrete approximations to the subjective distribution. To perform discretization we evaluated the distributions at the vertices of a regular 50×50 two-dimensional grid between $[-5, 5]$.

We computed the JS divergence between pairs of posterior mean subjective distributions, $\bar{\mathcal{P}}_i$ and $\bar{\mathcal{P}}_j$, corresponding to posterior distributions inferred from different datasets:

$$d_{i,j} = \sqrt{\mathrm{JS}\left[\bar{\mathcal{P}}_i\|\bar{\mathcal{P}}_j\right]} \tag{S15}$$

Crucially, even if the true subjective distributions underlying the two datasets were the same, our computed distance $d_{i,j}$ would not be exactly 0, because we perform inference on the basis of a finite, noisy data set. This baseline distance provides an approximate benchmark for the distances measured under different conditions when the subjective distributions underlying the datasets are not necessarily the same. To measure this baseline value empirically, we ran the inference algorithm separately for the two halves of the data collected for each subject and each task, using random and non-overlapping sets of $T = 500$ trials, and computed the distance between the resulting two posterior mean subjective distributions. This baseline distance, averaged across all subjects and both tasks, is shown in Figure 3A as a dashed line.

To visualize the result of inference across tasks and subjects (Fig. 3B) we performed multi-dimensional scaling [S14] on the full distance-matrix computed between all 20 estimated subjective distributions (10 subjects $\times$ 2 tasks each).

6.3 Assessing predictive performance

We evaluated the quality of our inferences by measuring the predictive performance of our model via cross-validation: we inferred its parameters on a subset of experimental data, and measured how accurately it

predicted responses in the held-out part of the data set. For this, we divided each data set (that is, one for each subject and each task) into $T_{\text{train}} = 700$ trials based on which we inferred the subjective distribution and other parameters of a subject, and $T_{\text{test}} = 300$ trials on which we used the inferred parameters to predict the subject's responses. To ensure uniform sampling of data over the whole course of the experiment, we divided the experiment into chunks of 10 subsequent trials, and subdivided these such that the training and test data included the first 7 and last 3 trials from all chunks, respectively.

6.3.1 Quantifying predictive performance

We used two metrics to quantify predictive performance. In Figure 4 we show the fraction of correct predictions. For this, we computed the probability of the subject choosing each of the possible responses in a trial using the corresponding ideal observer model with the inferred parameters, and then predicted the response that had the highest probability. We then computed the fraction of trials where the model's prediction matched the actual response of the subject.

Since subjects often do not give the same response even for the same stimuli (see section 6.5 for discussion on consistency) and our model actually predicts a probability distribution over all possible responses, rather than just a single response, we also used another metric of predictive performance that we call *probabilistic fraction correct* (Fig. S4). For this, we computed the predictive probability assigned by the model to the subject's actual response in each trial, and then took the geometric mean of these probabilities over the test set. (Note that this is equivalent to computing the likelihood of the model on test data.)

Probabilistic fraction correct is a more stringent metric of performance than the plain fraction correct metric because it requires the model to match the subject's full response distribution. It is therefore sensitive to the model being over-confident when making mistakes, and it is a good measure for evaluating how well a model is capable of representing the non-deterministic behavior of subjects. Similar to fraction correct, this more stringent measure showed that cognitive tomography had high predictive power even on a subject-by-subject basis both for within- or across-task predictions (Fig. S4).

6.3.2 Using Bayesian integration vs. point estimates for predictions

For predicting response probabilities for test stimuli, S^*, we computed the average prediction based on all possible parameter-combinations, $\theta = \{\mathcal{P}, \pi, \beta, \kappa, \Sigma_{\text{noise}}\}$, under the posterior distribution we inferred from our training data ($\{r^{(t)}, S^{*(t)}\}_{t=1}^{T_{\text{train}}}$, see also above):

$$\mathrm{P}\Big(r|S^*, \{r^{(t)}, S^{*(t)}\}_{t=1}^{T_{\text{train}}}\Big) = \int \mathrm{P}(r|S^*, \theta)\ \mathrm{P}\Big(\theta|\{r^{(t)}, S^{*(t)}\}_{t=1}^{T_{\text{train}}}\Big)\ \mathrm{d}\theta \tag{S16}$$

As in Figure. 2 we show the posterior mean subjective distributions (see section 6.1), for consistency, we also computed predictions based on these distributions. That is, rather than integrating out our uncertainty about the subjective distribution, $\mathcal{P}$, only in the final step of making predictions, as is statistically correct and done in Eq. S16, we first computed a point estimate over $\mathcal{P}$ and then made predictions based on that (but still integrated out our uncertainty about nuisance parameters, $\Omega = \{\pi, \beta, \kappa, \Sigma_{\text{noise}}\}$, only in the final step):

$$\mathrm{P}\Big(r|S^*, \{r^{(t)}, S^{*(t)}\}_{t=1}^{T_{\text{train}}}\Big) = \int \mathrm{P}\big(r|S^*, \bar{\mathcal{P}}, \Omega\big)\ \mathrm{P}\Big(\Omega|\{r^{(t)}, S^{*(t)}\}_{t=1}^{T_{\text{train}}}\Big)\ \mathrm{d}\Omega \tag{S17}$$

where $\bar{\mathcal{P}}$ is the posterior mean subjective distribution computed according to Eq. S12 (using only training data) .

Another alternative way to make predictions was to first calculate the maximum *a posteriori* (MAP) estimate for all the parameters, θ_{MAP}, and then make predictions based on that:

$$\mathrm{P}\Big(r|S^*, \{r^{(t)}, S^{*(t)}\}_{t=1}^{T_{\mathrm{train}}}\Big) \approx \mathrm{P}(r|S^*, \theta_{\mathrm{MAP}}) \tag{S18}$$

where

$$\theta_{\mathrm{MAP}} = \underset{\theta}{\mathrm{argmax}}\, \mathrm{P}\Big(\theta|\{r^{(t)}, S^{*(t)}\}_{t=1}^{T_{\mathrm{train}}}\Big) \tag{S19}$$

Figures 4 and S4 show predictions based on Bayesian integration (Eq. S16). As a control, we also computed the performance of approximate strategies for making predictions based on both MAP (Eq. S19, maximizing over samples of the posterior) and posterior mean estimates (Eq. S17). We found that predictive performances were essentially indistinguishable (Fig. S4E), which is due to the fact that our posteriors over parameters were sufficiently concentrated. As a consequence, plotting posterior mean subjective distributions for visualization (Figs. 2 and S1B-D, as described in section 6.1), provides a fair account of the distribution required for accurate predictions.

6.3.3 Predictions across tasks and across subjects

Our main goal was to test the task- and subject-specificity of subjective distributions. For this, we made predictions after swapping subjective distributions among tasks or subjects. However, even if subjective distributions are task- or subject-independent, the other 'nuisance' parameters can still be specific to subjects and tasks. Therefore, for making these kinds of predictions, we selectively swapped subjective distributions but not other decision parameters.

More specifically, for across-task predictions, we computed the parameters of each subject for making predictions in the familiarity task based on subjective distributions inferred from the odd-one-out task by the following procedure:

1. We inferred all parameters θ, including the subjective distribution $\mathcal{P}$, from data collected in the odd-one-out task.
2. We discarded all components but the subjective distribution, $\mathcal{P}$, from the resulting samples, and selected 10% of the samples of the subjective distribution (evenly spaced along the Markov chain) to carry over to trials of the familiarity task.
3. We re-inferred the remaining (nuisance) parameters, π, κ, β, and Σ_{noise}, from data collected in the familiarity task, conditioned on each sample subjective distribution we carried over from odd-one-out task.

Crucially, this procedure ensured that the inferred subjective distributions were not influenced by familiarity data.

For predicting behavior in the odd-one-out task based on data collected in the familiarity task, we had to take an additional difficulty into account. It is well known [S3] that in this task the likelihood defined by

Eqs. S3-S5 and S9 is degenerate in the sense that there are multiple configurations of the parameters $\mathcal{P}$, β, and Σ_{noise} that have the same likelihood. This means that subjective distributions can only be inferred up to certain invariances. This issue does not affect predictions in the familiarity task, but it would have an effect predicting odd-one-out responses based on familiarity data. To circumvent this potential confound, we computed the parameters of each subject for making predictions in the odd-one-out task based on subjective distributions inferred from the familiarity task by the following slightly more complex procedure:

1. We inferred θ from data collected in the odd-one-out task.
2. We discarded all parameters but β and Σ_{noise}, for which we retained 10% of the samples.
3. Conditioned on samples from the previous step we inferred the rest of the parameters ($\mathcal{P}$, π, and κ) from data collected in the familiarity task.
4. We discarded all parameters but the subjective distribution, $\mathcal{P}$, again for which we retained 10% of the samples.
5. Conditioned on subjective distributions from the previous step, we inferred the rest of the parameters from data collected in the odd-one-out task.

Again, crucially, this procedure ensured that the inferred subjective distributions were not influenced by odd-one-out data.

We also performed predictions across subjects. For this, we performed the following procedure for all pairs of subjects i and j separately for the two tasks:

1. We inferred all parameters from subject i's responses.
2. We discarded all parameters but the subjective distribution.
3. We inferred the rest of the parameters from subject j's data conditioned on the subjective distribution samples from subject i.

Across-subject predictive performance is at or below chance (Fig. S4B, D) demonstrating that subjective distributions are truly subject-specific.

6.4 Alternative models

We used a number of alternative models to control for different assumptions of cognitive tomography. The assumption that structural details of the inferred subjective distributions matter was tested by using a simple but statistically valid approximation of subjective distributions (section 6.4.1). The assumption that subjective distributions are relevant at all was tested by using a Gaussian process classifier which is a state-of-the-art discriminative learning algorithm that has no notion of subjective distributions (section 6.4.2). Finally, the assumption that subjects process stimuli in the two tasks in fundamentally different ways was tested by using alternative decision models for the odd-one-out task (section 6.4.3).

6.4.1 Moment matching

The subjective distributions we inferred and show in Figure 2 show complex, subject-specific structure, and seem to go beyond modeling means and simple linear correlations between dimensions. In order to test the degree to which this structural complexity is meaningful in that it contributes to explaining subjects' responses, we compared our predictions based on the full inferred subjective distributions against those derived from alternative subjective distributions that matched the first and second order moments of the original subjective distributions but contained no structure beyond that. For this control, we first computed the posterior mean subjective distributions for each task and subject (same as shown in Fig. 2), and then replaced each with the bivariate normal distribution matching its mean and covariance. We found that the moment matched model significantly under-performed predictions based on the full subjective distribution (Figs. 4C, D and S4B, D, F), which suggests that higher-order, complex structural features of subjective distributions carry meaningful information about subjects' responses.

6.4.2 Gaussian process classifier

We compared the predictive performance of our Bayesian model to a Gaussian process classifier (GPC) [S15]. This method directly learns a probabilistic input-output mapping from stimuli S to the subject's responses r, but it is completely ignorant of the task the subject tries to solve, or indeed the 'meaning' of responses. The input to the GPC consisted of 4 or 6 dimensional real vectors formed by concatenating the feature vectors of the two or three stimuli presented in each trial of the familiarity and odd-one-out tasks, respectively, and the output was the discrete response of the subject.

To make predictions in the familiarity task, we used a binary probit GPC model with automatic relevance determination kernel and used maximum likelihood to fit hyperparameters of the model. We used the open source GPML MATLAB library [S15]. For the odd-one-out task data we used a robust multiclass GPC [S16], and performed experiments using the source code made available by the authors of that algorithm.

For making predictions in the familiarity task we also tried a GPC using a kernel that was developed specifically to model preferential choice behavior [S17] and which thus had more prior information about the structure of the task the subjects performed. However, we found no significant improvement with this enhanced GPC compared to the standard GPC.

We found that cognitive tomography consistently outperformed the GPC (see subject-by-subject comparison in Fig. S4F, and group averages in Figs. 4 and S4B, D). However, one would expect the GP eventually to outperform any other method (ours included) in the limit of infinite data. We have conducted control simulations with large amounts of simulated data (not shown) and confirmed that this was indeed the case. The fact that cognitive tomography works better for limited amounts of data can be interpreted as an indication of the usefulness of the domain-specific prior knowledge we built in by using a subjective distribution-based formalism – ie. that it is quantitatively useful to assume that human behavior is based on using such subjective distributions.

6.4.3 Alternative ideal observer models for the odd-one-out task

An important assumption in our analysis is that subjects process stimuli in the two tasks, familiarity and odd-one-out, in substantially different ways. Indeed, in line with this assumption, our ideal observer models for the two tasks are markedly different. In particular, the odd-one-out task is not a widely studied task type, and to our knowledge we are the first to provide an ideal observer model for it. To assess whether human decisions were consistent with this model, and more generally, that they could not be explained by assuming that subjects performed the two tasks following similar decision rules, we compared our ideal observer model to two alternative models, *familiarity-min* and *familiarity-max*, that modeled subjects' behavior in the odd-one-out task essentially as if they were performing the familiarity task.

The *familiarity-max* model is analogous to our ideal observer-model for the familiarity task but with three instead of two alternatives presented. In this model, the subject evaluates the probability of each of the three stimuli being generated by their subjective distribution and prefers the stimulus that has the highest probability.

$$\mathrm{P}(r=i|S,\mathcal{P}) = (1-\kappa)\,\frac{\left[\pi_i \int \mathcal{O}(s_i;s_i^*)\,\mathcal{P}(s_i^*)\,\mathrm{d}s_i^*\right]^\beta}{\sum_j \left[\pi_j \int \mathcal{O}\big(s_j;s_j^*\big)\,\mathcal{P}\big(s_j^*\big)\,\mathrm{d}s_j^*\right]^\beta} + \frac{\kappa}{3} \tag{S20}$$

Under the *familiarity-min* model the subject evaluates the probability of each of the three stimuli being drawn from their subjective distribution and prefers the stimulus with the smallest probability. In this model, the subject tends to select the stimulus which is the 'oddest' on an absolute scale, rather than selecting the one which is odd when compared to the remaining two alternatives.

$$\mathrm{P}(r=i|S,\mathcal{P}) = (1-\kappa)\,\frac{\left[\pi_i \prod_{j\neq i} \int \mathcal{O}\big(s_j;s_j^*\big)\,\mathcal{P}\big(s_j^*\big)\,\mathrm{d}s_j^*\right]^\beta}{\sum_k \left[\pi_k \prod_{j\neq k} \int \mathcal{O}\big(s_j;s_j^*\big)\,\mathcal{P}\big(s_j^*\big)\,\mathrm{d}s_j^*\right]^\beta} + \frac{\kappa}{3} \tag{S21}$$

$$= (1-\kappa)\,\frac{\pi_i^\beta \left[\int \mathcal{O}(s_i;s_i^*)\,\mathcal{P}(s_i^*)\,\mathrm{d}s_i^*\right]^{-\beta}}{\sum_j \pi_j^\beta \left[\int \mathcal{O}\big(s_j;s_j^*\big)\,\mathcal{P}\big(s_j^*\big)\,\mathrm{d}s_j^*\right]^{-\beta}} + \frac{\kappa}{3} \tag{S22}$$

We found that the original ideal observer model for the odd-one-out task outperformed both the *familiarity-min* and the *familiarity-max* models in both within-task and across-task predictions (Fig. S4B, D).

6.5 Consistency and predictability

None of the models we implemented, including cognitive tomography, could predict subjects' responses with 100% accuracy (Figs. 4 and S4). This could be a deficiency of these models, or it could be an inevitable consequence of subjects' noisy behavior. Intuitively, if a subject deterministically gives the same response to the same stimuli each time, it should be possible to predict their responses with 100% accuracy. Conversely, if the subject's responding is uniformly random and independent of stimuli, no predictive model could surpass chance level. Therefore, we measured subjects' consistency, the fraction of trials with identical responses to the same stimuli, and based on this consistency score we derived a model-free expected upper bound on the predictability of their behavior. The following results closely follow those found in Ref. [S18], we only include them here for completeness.

Note that the calculations below assume that both consistency and predictability (fraction correct) can be measured exactly (continuous integrals over stimulus space in Eqs. S24-S25), as if we used infinitely many trials to estimate them. Since, by necessity, these quantities must be measured using a finite set of trials in experiments, both quantities are plotted with confidence intervals in Fig. 4E-F.

6.5.1 Two-alternative choice tasks (e.g. familiarity)

For this analysis we only assume that subjects' responding is independent given the stimuli presented in each trial and the corresponding response probabilities. Let us denote the probability of the subject's most probable response for a given set of stimuli S by $p(S)$. Thus, by definition

$$\frac{1}{2} \leq p(S) \leq 1 \tag{S23}$$

The subject's predictability, f^* is defined as the best predictive performance achievable by any predictor. Predictive performance is measured by the expected fraction of correct predictions, assuming sets of stimuli are sampled from $P_\mathrm{s}(S)$. The best predictive performance is achieved by a predictive model using MAP estimation based on $p(S)$, which always selects the subject's most probable response for each set of stimuli. In expectation, such a predictor achieves the following performance:

$$f^* = \int \mathrm{d}S \, P_\mathrm{s}(S) \; p(S) \tag{S24}$$

Unfortunately, it is impossible to estimate f^* directly from data, without assuming a particular value or form for $p(S)$. We will therefore focus on deriving an upper bound on f^* that depends on quantities that can be estimated from experimental data.

A key quantity in our analysis is a subject's consistency, c, which is assessed by having a number of trial-pairs repeating exactly the same set of stimuli, and measuring the fraction of trial-pairs out of these on which the subject's response was identical. Using our formalism, the average probability with which a subject gives the same answer in two trials using the same set of stimuli can be expressed as

$$c = \int \mathrm{d}S \, P_\mathrm{s}(S) \left[p(S)^2 + (1 - p(S))^2 \right] \tag{S25}$$

This quantity depends on the subject's response probabilities $p(S)$ and on the distribution $P_{\mathrm{s}}(S)$ from which sets of stimuli are sampled on consistency trials – and which we assume is the same as the distribution of stimulus sets used on all other trials.

It is easy to see that predictability is lower bounded by consistency:

$$f^* \geq c \tag{S26}$$

Importantly, it is also possible to compute an upper bound on f^*, and consequently on the predictive performance of any model, using the consistency c.

We will use $\mathbb{E}[\ldots]$ to denote expectation under the stimulus set distribution that is $\int \mathrm{d}S\, P_{\mathrm{s}}(S)\ldots$ and rewrite f^* and c as

$$f^* = \mathbb{E}[p(S)] \tag{S27}$$

and

$$\begin{aligned} c &= \mathbb{E}\Big[p(S)^2 + (1-p(S))^2\Big] && \text{(S28)}\\ &= 2\,\mathbb{E}\Big[p(S)^2\Big] - 2\,\mathbb{E}[p(S)] + 1 && \text{(S29)}\\ &= 2\,\Big(\mathrm{Var}[p(S)] + \mathbb{E}[p(S)]^2\Big) - 2\,\mathbb{E}[p(S)] + 1 && \text{(S30)}\\ &= 2\,\Big(\mathrm{Var}[p(S)] + f^{*2}\Big) - 2\,f^* + 1 && \text{(S31)}\\ &\geq 2\,f^{*2} - 2\,f^* + 1 && \text{(S32)} \end{aligned}$$

This leaves us an *upper bound* on f^*:

$$f^* \leq \frac{1+\sqrt{2\,c-1}}{2} = f^*_{\mathrm{max}} \tag{S33}$$

We note that we also obtain a lower bound, $f^* \geq \frac{1-\sqrt{2\,c-1}}{2}$, but it is looser than the consistency c, that we derived in Eq. S26, and as such it can be ignored.

In summary, knowing the subject's consistency c we have both a lower and an upper bound on their predictability f^*:

$$c \leq f^* \leq \frac{1+\sqrt{2\,c-1}}{2} = f^*_{\mathrm{max}} \tag{S34}$$

The upper bound is shown in Figure 4E.

6.5.2 Three-alternative choice tasks (e.g. odd-one-out)

The same reasoning applies when the subject can choose from three alternative responses, such as in the odd-one-out task.

We start with the same assumptions as in the previous section. However, since there are now three options in each trial, we will denote the probability of the response with the highest probability (preferred response) by $p_{\mathrm{H}}(S)$, and the probability of the response with the lowest probability (dispreferred response) by $p_{\mathrm{L}}(S)$.

(The probability of the third response is $1 - p_\mathrm{H}(S) - p_\mathrm{L}(S)$). Thus, to be consistent with their definitions, these quantities must obey the following constraints:

$$\frac{1}{3} \leq \quad p_\mathrm{H}(S) \quad \leq 1 \tag{S35}$$

$$\max\left(0, 1 - 2\, p_\mathrm{H}(S)\right) \leq \quad p_\mathrm{L}(S) \quad \leq \frac{1 - p_\mathrm{H}(S)}{2} \tag{S36}$$

Now, f^* is obtained just as before:

$$f^* = \mathbb{E}[p_\mathrm{H}(S)] \tag{S37}$$

Consistency, c can also be estimated as before, as the fraction of trial-pairs the subject selected the same response when the same stimulus set was presented. Its formula becomes slightly more involved:

$$c = \mathbb{E}\Big[p_\mathrm{H}(S)^2 + p_\mathrm{L}(S)^2 + (1 - p_\mathrm{H}(S) - p_\mathrm{L}(S))^2\Big] \tag{S38}$$

It is easy to show that for a given value of $p_\mathrm{H}(S)$, c is minimized when $p_\mathrm{L}(S)$ is at its maximum, that is when

$$p_\mathrm{L}(S) = \frac{1 - p_\mathrm{H}(S)}{2} \tag{S39}$$

Substituting this back into the formula for c, Eq. S38, we obtain

$$c \geq \mathbb{E}\left[p_\mathrm{H}(S)^2 + 2\left(\frac{1 - p_\mathrm{H}(S)}{2}\right)^2\right] \tag{S40}$$

$$= \frac{3}{2}\,\mathbb{E}\Big[p_\mathrm{H}(S)^2\Big] - \mathbb{E}[p_\mathrm{H}(S)] + \frac{1}{2} \tag{S41}$$

$$= \frac{3}{2}\left(\mathrm{Var}[p_\mathrm{H}(S)] + \mathbb{E}[p_\mathrm{H}(S)]^2\right) - \mathbb{E}[p_\mathrm{H}(S)] + \frac{1}{2} \tag{S42}$$

$$= \frac{3}{2}\left(\mathrm{Var}[p_\mathrm{H}(S)] + f^{*2}\right) - f^* + \frac{1}{2} \tag{S43}$$

$$\geq \frac{3}{2}\, f^{*2} - f^* + \frac{1}{2} \tag{S44}$$

Rearranging the inequality, we obtain the following upper bound f^*_max:

$$f^* \leq \frac{1 + \sqrt{6\,c - 2}}{3} = f^*_\mathrm{max} \tag{S45}$$

Qualitatively, this upper bound is very similar to the upper bound found in the two alternative-choice case (Fig. 4F).

Consistencies and predictabilities for the two task-types are shown in Figure 4E-F. Importantly, the predictability bound f^*_max in both tasks is independent from the model we use to make predictions, and even the details of the task subjects are performing. It applies to any model and any task in which subjects choose from two or three alternatives and their responding is assumed to be independent given the stimuli and corresponding response probabilities. Also note that the bound is relatively loose because it assumes that the variance of maximal response probabilities across trials (or stimulus sets) is zero. In the three-alternative choice case, it is even looser because the probability of dispreferred responses is also assumed to take the highest possible value in all trials (i.e. for all stimulus sets). Considering the looseness of these bounds, it is all the more notable that our predictive performance often comes remarkably close to them.

6.6 Subject-specific chance levels

The performance of predictive models was compared to the chance level, which in case there are R possible outcomes to predict, is usually taken to be $1/R$ (Figs. 4 and S4E-F). This is the predictive performance of a naive strategy, that randomly picks each possible outcome with equal probability, and any sensible method should surpass it.

However, as our subjects did not choose each response with equal probability during the experiment, there is a more stringent chance level which is specific to each subject. We can consider the performance of the best predictor that ignores the stimuli presented to the subject, but exploits imbalance in their responses.

If stimulus sets are sampled from $P_{\mathrm{s}}(S)$, and $p_i(S)$ denotes the probability of the subject choosing response i when stimulus set S is presented, then the subject's average probability of choosing response i, $\bar{p}_i$ is

$$\bar{p}_i = \int \mathrm{d}S \, P_{\mathrm{s}}(S) \; p_i(S) \tag{S46}$$

Under the fraction correct evaluation, the best predictor of the subject's responses that ignores the stimuli presented always predicts response $i^* = \mathrm{argmax}_i \, \bar{p}_i$. This predictor achieves the following fraction correct level:

$$f^- = \int \mathrm{d}S \, P_{\mathrm{s}}(S) \sum_{i=1}^{R} p_i(S) \; \delta_{i,i^*} = \max_i \bar{p}_i \tag{S47}$$

We can see that $1/R \leq f^- \leq 1$, therefore when subjects choose, on average, each response uniformly, the subject-specific chance level f^- reduces to the classical $1/R$ level.

Under the probabilistic fraction correct evaluation, the best predictor that ignores the stimulus presented estimates the subject's probability of choosing response i to any stimulus as $\bar{p}_i$. This yields the following probabilistic fraction correct level:

$$f^-_{\text{prob}} = e^{\int \mathrm{d}S \, P_{\mathrm{s}}(S) \sum_{i=1}^{R} p_i(S) \, \log \bar{p}_i} = e^{-\mathbb{H}[\{\bar{p}_i\}]}, \tag{S48}$$

where $\mathbb{H}[\cdot]$ denotes Shannon's entropy. Again, it can be shown that $1/R \leq f^-_{\text{prob}} \leq 1$ and that f^-_{prob} reduces to the traditional chance level $1/R$ if and only if the subject chooses each response with the same probability on average. Figure S4A-D shows subject-specific chance levels under the probabilistic fraction correct evaluation.

Supplemental References

S1. Paysan, P., Knothe, R., Amberg, B., Romdhani, S. & Vetter, T. A 3D face model for pose and illumination invariant face recognition. in *Sixth IEEE International Conference on Advanced Video and Signal Based Surveillance* , 296–301, (2009).

S2. Stocker, A.A. & Simoncelli, E.P. Noise characteristics and prior expectations in human visual speed perception. *Nat. Neurosci.* **9**, 578–585 (2006).

S3. Sanborn, A.N. & Griffiths, T.L. Markov chain Monte Carlo with people. in *Advances in Neural Information Processing Systems 20* (eds. Platt, J.C., Koller, D., Singer, Y. & Roweis, S.) 1265–1272 (MIT Press, 2008).

S4. Calder, A.J. & Young, A.W. Understanding the recognition of facial identity and facial expression. *Nat Rev Neurosci* **6**, 641–51 (2005).

S5. Fiser, J., Berkes, B., Orbán, G. & Lengyel, M. Statistically optimal perception and learning: from behavior to neural representations. *Trends Cogn. Sci.* **14**, 119–130 (2010).

S6. Houlsby, N., Huszár, F., Ghahramani, Z. & Lengyel, M. Bayesian active learning for classification and preference learning. *arXiv*, 1112.5745 (2011).

S7. Huszár, F., Noppeney, U. & Lengyel, M. Mind reading by machine learning: a doubly Bayesian method for inferring mental representations. in *Proceedings of the Thirty-Second Annual Conference of the Cognitive Science Society* 2810–2815 (2010).

S8. Noreen, D.L. Optimal decision rules for some common psychophysical paradigms. in *Mathematical Psychology and Psychophysiology* Vol. 13 (ed. Grossberg, S.), Vol. 13, 237–280 (Erlbaum & American Mathematical Society, Hillsdale, NJ & Providence, RI, 1981).

S9. Kemp, C., Bernstein, A. & Tenenbaum, J.B. A generative theory of similarity. in *Proceedings of the Twenty-Seventh Annual Conference of the Cognitive Science Society* 1132–1137 (2005).

S10. Shepard, R.N. Toward a universal law of generalization for psychological science. *Science* **237**, 1317–1323 (1987).

S11. Wichmann, F.A. & Hill, N.J. The psychometric function: I. fitting, sampling, and goodness of fit. *Percept Psychophys* **63**, 1293–313 (2001).

S12. Gupta, A. & Nagar, D. *Matrix Variate Distributions* (Chapman & Hall/CRC, 2000).

S13. Neal, R.M. MCMC using Hamiltonian dynamics. in *Handbook of Markov Chain Monte Carlo* (eds. Brooks, S., Gelman, A. & Meng, X.L.) 113–162 (Chapman & Hall/CRC, 2010).

S14. Borg, I. & Groenen, P.J.F. *Modern Multidimensional Scaling* (Springer Verlag, 2005).

S15. Rasmussen, C.E. & Williams, C.K.I. *Gaussian Processes for Machine Learning* (MIT Press, 2005).

S16. Hernández-Lobato, D., Hernández-Lobato, J.M. & Dupont, P. Robust multi-class Gaussian process classification. in *Advances in Neural Information Processing Systems (NIPS)* (eds. Shawe-Taylor, J., Zemel, R., Bartlett, P., Pereira, F. & Weinberger, K.) 280–288 (MIT Press, 2011).

S17. Chu, W. & Ghahramani, Z. Preference learning with Gaussian processes. in *Proceedings of the Twenty-Second International Conference on Machine Learning* 137–144 (ACM, 2005).

S18. Neri, P. & Levi, D.M. Receptive versus perceptive fields from the reverse-correlation viewpoint. *Vision Res* **46**, 2465–74 (2006).
